# The Impact of Chemotherapy on Gonadal Function in Female Patients with Hodgkin and Non-Hodgkin Lymphoma: A Comprehensive Analysis of Hormonal Kinetics and Implications for Fertility and Contraceptive Planning

**DOI:** 10.3390/cancers18172855

**Published:** 2026-09-03

**Authors:** Angeliki N. Georgopoulou, Theodoros P. Vassilakopoulos, Andreas Giannakou, Neoklis A. Georgopoulos, Sophia Kalantaridou, Konstantinos Keramaris, Eleni Lalou, Eleni Loukari, Konstantinos Konstantopoulos, Marina P. Siakantaris, Anastasia Kopsaftopoulou, Chrysovalanto Chatzidimitriou, Maria Arapaki, Marina Belia, Iliana Konstantinou, Ioannis Asimakopoulos, Irene Mammali, Maria K. Angelopoulou

**Affiliations:** 1 Department of Hematology and Bone Marrow Transplantation, School of Medicine, National and Kapodistrian University of Athens, Laikon General Hospital, 11527 Athens, Greece; theopvass@hotmail.com (T.P.V.); andersgian@gmail.com (A.G.); kosker@med.uoa.gr (K.K.); eleni_lalou@yahoo.gr (E.L.); eleniloukari@gmail.com (E.L.); kkonstan@med.uoa.gr (K.K.); siakantaris@gmail.com (M.P.S.); an.kopsaftopoulou@gmail.com (A.K.); cchatzidimitriou@hotmail.com (C.C.); arapaki_m@hotmail.gr (M.A.); beliamarina@hotmail.com (M.B.); eliankon@hotmail.com (I.K.); jv.asimakopoulos@gmail.com (I.A.); mkangelop@gmail.com (M.K.A.); 2 Department of Internal Medicine, Division of Endocrinology, University of Patras Medical School, 26110 Patras, Greece; neoklisgeorgo@gmail.com (N.A.G.); irenemamali@gmail.com (I.M.); 3 B Obstetrics and Gynecology Department, Attikon General Hospital, National and Kapodistrian University of Athens, 12462 Athens, Greece; sophiakalantaridou@gmail.com

**Keywords:** Hodgkin lymphoma, primary mediastinal B-cell lymphoma, chemotherapy, fertility, gonadal insufficiency, fertility preservation, anti-Müllerian hormone, follicle-stimulating hormone

## Abstract

**Background/Objectives**: Hodgkin lymphoma (HL) and primary mediastinal B-cell lymphoma (PMBCL) affect young women, with cure rates exceeding 80%. Treatment-related gonadal insufficiency is recognized for its impact on fertility, yet fertility preservation remains underutilized, and prospective data are limited. The aim of this study is to prospectively evaluate gonadal function in women ≤40 years old with lymphoma undergoing chemotherapy. **Methods**: Ovarian reserve and endocrine ovarian function were evaluated by sequential measurements of follicle-stimulating hormone (FSH), luteinizing hormone (LH), anti-Müllerian hormone (AMH), progesterone, and estradiol at diagnosis, during, and after chemotherapy. **Results**: 81 female patients ≤40 years old were enrolled, including 53 with HL and 28 with NHL (16 with PMBCL). HL patients had significantly lower AMH values for their respective age group compared to all other diagnoses (*p* = 0.05), which was more striking for patients ≤30 years old (*p* = 0.039), indicating pre-existing reduced ovarian reserve in HL. In HL, both FSH and AMH levels indicate gonadal dysfunction for at least six months post-chemotherapy, with AMH serving as a more sensitive biomarker than FSH. For PMBCL patients treated with R-DA-EPOCH, AMH suppression was noticed, with no evidence of recovery up to 18 months post-treatment. At all time points, the PMBCL patients had significantly lower AMH values compared to the HL patients (AMH6: *p* = 0.03, AMH12 and AMH18: *p* = 0.05). AMH emerged as the most sensitive marker of ovarian damage, with pretreatment levels <7 pmol/L predicting impaired ovarian reserve in HL patients. **Conclusions:** For HL, the pretreatment cut-off of 7 pmol/L could discriminate patients with ovarian insufficiency post-treatment, whereas no statistically significant predictive association was identified in PMBCL. These findings require validation in larger cohorts.

## Simple Summary

Young women with Hodgkin lymphoma (HL) and primary mediastinal B-cell lymphoma (PMBCL) have excellent survival rates, making preservation of reproductive function an important aspect of long-term care. However, prospective data on chemotherapy-induced ovarian damage have remained limited. In this prospective study, ovarian function was serially evaluated before, during, and after chemotherapy using established hormonal markers. Anti-Müllerian hormone (AΜH) emerges as the most sensitive and reliable marker of gonadal function, with a threshold of 7 pmol/L being predictive of impaired ovarian reserve in HL patients. Women with HL demonstrated evidence of reduced ovarian reserve prior to treatment, and pretreatment AMH concentrations identified patients at increased risk of persistent ovarian insufficiency following chemotherapy. In contrast, patients with PMBCL treated with dose-adjusted EPOCH-R experienced more sustained ovarian impairment, and no pretreatment biomarker reliably predicted ovarian recovery. These findings characterize treatment-related changes in ovarian reserve and may support individualized pretreatment reproductive counseling in young women receiving chemotherapy for lymphoma.

## 1. Introduction

Among hematologic malignancies, Hodgkin lymphoma (HL) and primary mediastinal large B-cell lymphoma (PMBCL) predominantly affect young adults. These conditions are considered curable in over 80% of this population with current treatment approaches [1,2,3]. As cure rates increase, long-term treatment-related complications are receiving growing attention. These mainly include cardiomyopathy linked to anthracyclines and/or mediastinal radiotherapy and secondary malignancies such as lung and breast cancers due to radiotherapy or secondary leukemias [4,5]. A notable long-term complication is potential infertility and gonadal insufficiency, which carry significant psychological and social consequences, particularly for young females [6], due to the time-sensitive nature of fertility preservation strategies.

ABVD (adriamycin, bleomycin, vinblastine, dacarbazine) and R-CHOP (rituximab, cyclophosphamide, doxorubicin, vincristine, prednisone) or R-DA-EPOCH (rituximab, prednisolone, dose-adjusted cyclophosphamide, doxorubicin, etoposide, vincristine) regimens, used to treat HL and PMBCL, respectively, are generally perceived as low-risk for gonadotoxicity, largely based on retrospective childbirth data [7]. Conversely, BEACOPP-escalated (bleomycin, etoposide, adriamycin, cyclophosphamide, vincristine, procarbazine, prednisolone) has been associated with reduced ovarian reserve in advanced-stage HL, with the cumulative number of cycles playing a key role [8]. Most of the fertility data in HL derive from survivors who participated in clinical trials by the German Hodgkin Study Group (GHSG) and the European Organization for Research and Treatment of Cancer (EORTC). R-DA-EPOCH (rituximab, prednisolone, dose-adjusted cyclophosphamide, doxorubicin, etoposide, vincristine) has been recently adopted by many centers for PMBCL, with very limited information on its impact on gonadal function [9,10,11,12].

In Greece, fertility preservation presents additional challenges. The logistics of oocyte or ovarian tissue collection—requiring hormonal stimulation, time, and invasive procedures—are often impossible to implement during the narrow pretreatment window [13,14]. Moreover, multidisciplinary fertility preservation teams, essential for timely intervention, are limited even in academic centers. Therefore, it is crucial for clinicians to promptly identify patients who may not require standard fertility preservation techniques.

The aim of the present longitudinal, prospective study is to evaluate gonadal function in young women undergoing standard treatment for HL and NHL conducted at a single academic institution.

## 2. Materials and Methods

### 2.1. Patients and Study Design

This prospective study assesses gonadal function in female patients with HL, PMBCL, diffuse large B-cell lymphoma (DLBCL), follicular lymphoma (FL), and Burkitt lymphoma (BL). We included patients aged 16–40 years with biopsy-proven lymphoma receiving first-line therapy. Patients had to be in continuous remission for at least one year following first-line treatment and should not have received any additional chemotherapy (i.e., patients who relapsed went off study upon 2nd line treatment). Patients with a medical history of malignancies, HIV infection, and those who were pregnant or lactating were excluded. Treating physicians obtained written informed consent from all participants, following a comprehensive discussion, both for blood sampling at predefined time points and for the use of diagnostic and laboratory data. We treated and followed all patients at the Department of Haematology, National and Kapodistrian University of Athens. This study was designed in accordance with the Declaration of Helsinki and was approved by the Institutional Review Board (IRB) of Laikon General Hospital.

### 2.2. Assessment of Ovarian Function and Reproductive Outcomes

Gonadal function was evaluated before, during, and after treatment. Patients’ data collected included past medical history, menstrual cycle characteristics, time to resumption of menstrual activity, disease features, chemotherapy regimens (including number of cycles, schedules, and doses), and radiotherapy details (including duration, dose, and field). A normal menstrual cycle was defined as a cycle length of 24–38 days, menstrual bleeding lasting ≤8 days, and regular cyclicity with minimal intercycle variability [14].

### 2.3. Hormonal Analysis

Whole blood samples were collected without anticoagulant for hormonal analysis at 6 predefined intervals, namely, (1) at diagnosis, before the commencement of chemotherapy (t0); (2) mid-treatment (t1); (3) at the end of treatment (t2); (4) at six months post-treatment (t6); (5) at 12 months post-treatment (t12) and (6) at 18 months post-treatment (t18). Post-collection, samples were centrifuged at 3000 rpm for 10 min, and serum was aliquoted and stored at −20 °C until analysis. Hormonal measurements were conducted using standardized commercial reagents: the ANACHEM Pico AMH ELISA kit (Ansh Labs, Webster, TX, USA) for anti-Müllerian hormone (AMH) and standardized serum assays for follicle-stimulating hormone (FSH), luteinizing hormone (LH), progesterone (PG), and estradiol (E2) [heterogenic, noncompetitive chemiluminescent immunometric assays, Elecsys-LH, Elecsys-FSH, Elecsys-Estradiol-II (cobas e601 Roche Hitachi)].

According to the assay kit employed in our study, the normal median AMH values (and 2.5th–97.5th percentiles) in healthy women were 28.6 pmol/L (8.7–83.6) [ages 20–24 years], 23.6 pmol/L (6.35–70.3) [ages 25–29 years], 20.0 pmol/L (4.11–58) [ages 30–34 years], 14.2 pmol/L (1.05–53.5) [ages 35–39 years], 6.29 pmol/L (0.193–39.1) [ages 40–44 years] [15].

### 2.4. Statistical Analysis

All data were entered into an electronic database and analyzed using IBM SPSS Statistics version 25 for Windows (IBM Corp., Armonk, NY, USA). The distribution of continuous variables was assessed using the Shapiro–Wilk test. Variables with non-normal distributions are presented as medians and ranges (minimum–maximum), whereas categorical variables are presented as frequencies and percentages. Comparisons of continuous variables between two independent groups were performed using the Mann–Whitney U test, while comparisons across more than two independent groups were performed using the Kruskal–Wallis test. Associations between categorical variables were evaluated using Pearson’s chi-square test. Correlations between continuous variables were assessed using Spearman’s rank correlation coefficient.

For the longitudinal hormonal analyses, hormone levels at each follow-up assessment were compared separately with their corresponding pretreatment baseline values using the two-sided Wilcoxon signed-rank test. These comparisons were performed for AMH, FSH, LH, estradiol, and progesterone within the HL cohort, the overall NHL cohort, and the subgroup of patients with PMBCL treated with R-DA-EPOCH. Follow-up assessments were conducted at mid-treatment, treatment completion, and 6, 12, and 18 months after treatment. Each comparison included only patients with measurements available at both baseline and the corresponding follow-up assessment. Missing values were not imputed; consequently, the number of paired observations varied across comparisons. No adjustment for multiple comparisons was applied, and the resulting *p*-values should therefore be interpreted as exploratory. All tests were two-sided, and *p*-values < 0.05 were considered statistically significant.

The evaluated reproductive outcomes included menstrual-cycle recovery and time to recovery, and hormonal markers of ovarian function and ovarian reserve. The principal between-group analysis focused on patients with HL and those with PMBCL treated with R-DA-EPOCH. Given the age-related variation in ovarian reserve, additional exploratory subgroup analyses were performed among patients aged 16–30 years [16,17]. Because of the limited number of patients with available follow-up measurements and the sparse outcome data within diagnostic subgroups, no multivariable adjustment for potential confounders was performed, as this would have produced unstable and potentially overfitted estimates. An exploratory post hoc threshold analysis was conducted within the HL cohort. Candidate pretreatment AMH thresholds were examined in relation to age-adjusted low AMH at 12 months after treatment. A threshold of 7 pmol/L was selected based on the separation observed within the present cohort. This threshold was not prespecified, and the ROC curve, AUC, sensitivity, and specificity are provided. Therefore, it should be considered hypothesis-generating rather than a validated clinical cut-off. Although internal validation via bootstrap resampling was not performed due to the small sample size, we emphasize that this threshold should be independently validated in larger cohorts.

## 3. Results

### 3.1. Patient Characteristics

Eighty-one women participated in this study: 53 with HL and 28 with NHL. Patient characteristics are summarized in Table 1. The median age of HL patients was 28 years, and the majority (74%) had stage II disease. Forty-seven patients (89%) received the standard ABVD regimen, whereas six (11%) received BV-AVD (brentuximab vedotin-AVD). Among the 28 women with NHL, who had a median age of 29 years, 16 patients (57%) were diagnosed with PMBCL and received dose-adjusted R-EPOCH, nine patients (32%) with DLBCL and were treated with R-CHOP-21, two (7%) with follicular lymphoma (FL) and were treated with anti-CD20 antibody plus bendamustine, and one patient (4%) with Burkitt lymphoma and received the GMALL protocol for Burkitt.

Hormonal measurements were available for 81 patients at baseline. Follow-up measurements were available for 64 patients (79.0%) at mid-treatment, 54 (66.7%) at treatment completion, 40 (49.4%) at 6 months, 40 (49.4%) at 12 months, and 30 (37.0%) at 18 months. The number of patients contributing data to each analysis is also reported in parentheses in Table A1.

A similar proportion of HL (24%) and NHL (22%) patients had given birth before diagnosis. Abnormal menstrual cycles before treatment were reported in 16% of HL vs 14% of NHL patients. Baseline patient characteristics did not differ significantly between the two treatment groups, except for GnRH analogue administration during chemotherapy, which was more common among patients with NHL than among those with HL (44% vs. 12%, *p* = 0.01), and oocytes cryopreservation, which was more frequently performed in HL patients (30% vs. 12% in NHL, *p* = 0.04). In addition, pretreatment CRP levels differed significantly among diagnoses (*p* < 0.001), with the highest values observed in HL (median: 31.03 mg/L; range: 0.14–301) and PMBCL (median: 28.5 mg/L; range: 0.52–115). Although no statistically significant difference was noted between HL and PMBCL overall, when the analysis was restricted to patients ≤ 30 years of age, a trend towards higher CRP levels in HL compared to PMBCL was observed (*p* = 0.556).

All patient characteristics are summarized in Table 1.

**Figure 1:**
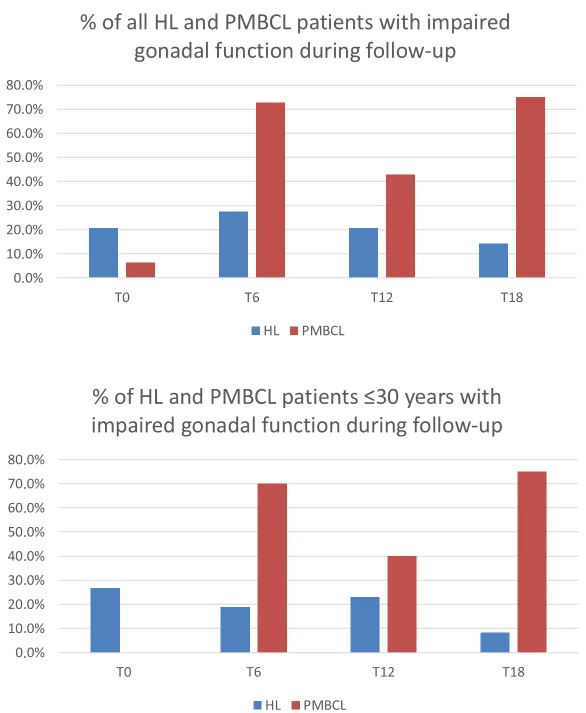
Percentage of HL and PMBCL patients with impaired gonadal function during follow-up. Note: T0 = baseline, T6 = 6 months post-treatment, T12 = 12 months post-treatment, T18 = 18 months post-treatment. Abbreviations: HL: Hodgkin lymphoma, PMBCL: primary mediastinal B-cell lymphoma.

All 81 patients remained under clinical follow-up. The number of patients with hormone measurements available at each scheduled assessment, stratified by diagnostic subgroup, is presented in Figure 1. Reduced sample availability at later assessments reflected the absence of blood samples within the prespecified sampling windows rather than loss to clinical follow-up. See the temporal evolution of hormonal measurements in Table 2.

### 3.2. Baseline Ovarian Reserve

Baseline AMH concentration, as a continuous variable obtained at disease diagnosis, did not differ significantly across diagnostic groups, either among the entire cohort, between HL and PMBCL patients, or within the subgroup of patients aged ≤30 years. However, when categorized according to normal values for age, marked differences emerged: Surprisingly, at baseline, before treatment, HL patients had significantly lower AMH values for their respective age group compared to all other diagnoses [11/53 (21%) vs. 1/25 (4%), *p* = 0.05]. This difference was even more pronounced among patients ≤30 years of age, where 8/30 (27%) of HL patients had subnormal AMH levels for their age group versus none (0/13) among patients with all other diagnoses (*p* = 0.039), indicating pre-existing reduced ovarian reserve in HL. Furthermore, there was a trend for a negative correlation between AMH and CRP in women with HL aged ≤ 30 years (*p* = 0.173). No statistically significant associations were observed between baseline AMH and disease stage or the presence of B symptoms. However, these analyses may have been limited by the small sample size.

### 3.3. AMH Kinetics During and After Chemotherapy

During chemotherapy, both groups experienced a rapid and substantial decline in median AMH levels. However, different patterns of AMH kinetics were observed according to diagnoses and respective treatment regimens.

In HL, AMH showed a significant decrease by mid-treatment (AMH1: 1.05 pmol/L; *p* < 0.001 vs. baseline) and remained suppressed at treatment completion (AMH2: 2.82 pmol/L; *p* < 0.001 vs. baseline). Recovery was observed at 6 months (AMH6: 9.92 pmol/L; *p* = 0.1 vs. baseline), which was sustained at 12 and 18 months (AMH12: 9.90 pmol/L; AMH18: 12.3 pmol/L; *p* = ns vs. baseline). All patients followed the expected AMH kinetics observed across the cohort.

In NHL patients, the decline in median AMH values was equally sharp, but recovery was slower and less complete. Among patients with NHL, ovarian-function trajectories were evaluated according to diagnosis and treatment regimen. Because of the small numbers of patients with DLBCL (n = 9), follicular lymphoma (n = 2), and Burkitt lymphoma (n = 1), their results are presented descriptively, and no formal comparisons were performed. The principal comparative analysis therefore focused on patients with HL and those with PMBCL treated with R-DA-EPOCH. In DLBCL and FL patients, AMH started to recover at 12 months (AMH12: 8.96 pmol/L), and this recovery was sustained at 18 months (AMH18: 10.29 pmol/L); AMH values and did not differ significantly from pretreatment values (AMH0: 14.76 pmol/L).

In HL patients, median AMH concentrations in those with versus without GnRH analogue exposure were 6.94 (n = 6) vs. 11.31 (n = 47) pmol/L at baseline and 14.43 (n = 3) vs. 9.19 (n = 26) pmol/L at 6 months. In the PMBCL/gray-zone cohort treated with R-DA-EPOCH, corresponding values were 14.23 (n = 10) vs. 15.83 (n = 5) pmol/L at baseline and 5.10 (n = 5) vs. 2.34 (n = 6) pmol/L at 6 months. These findings are descriptive; small subgroups and limited later measurements, including no 12- or 18-month AMH data in GnRH-exposed HL patients, prevent firm conclusions regarding the effect of GnRH analogues on AMH recovery.

Contrary to HL, DLBCL and FL, PMBCL patients experienced the most severe gonadal damage: AMH dropped to nearly undetectable levels by mid-treatment (AMH1: 0.16 pmol/L; *p* < 0.001), remained extremely low at the end of the treatment (AMH2: 0.22 pmol/L; *p* = 0.003 vs. AMH0: 15.83 pmol/L), continued to be suppressed at 6 months (AMH6: 4.51 pmol/L; *p* = 0.005 vs. baseline), and remained without significant improvement even at 18 months (AMH12: 1.32 pmol/L, AMH18: 3.91 pmol/L). All these post-treatment values were significantly lower than the baseline AMH value of 15.83 pmol/L).

### 3.4. Different Patterns of Post-Treatment Gonadal Function in HL vs. PMBCL

From this point on, results focus on HL (N = 53) and PMBCL (N = 16) patients, who represent the vast majority of the studied population and, even more specifically, on patients ≤ 30 years of age.

When analyzing AMH as a continuous variable, at all post-treatment time points, PMBCL patients had significantly lower AMH values compared to HL patients (AMH6: *p* = 0.03, AMH12 and AMH18: *p* = 0.05). The median AMH levels for HL were 9.92 pmol/L, 9.9 pmol/L, and 12.32 pmol/L at 6, 12, and 18 months, respectively, while for PMBCL the corresponding values were 4.51 pmol/L, 1.32 pmol/L, and 3.91 pmol/L, respectively.

Among those aged ≤ 30 years, this difference was even more pronounced (AMH6: *p* = 0.001; AMH12: *p* = 0.019; AMH18: *p* = 0.004). The median AMH levels for this subgroup aged ≤30 years were 14.89 pmol/L, 18.45 pmol/L, and 20.41 pmol/L at 6, 12, and 18 months, respectively for HL, while for PMBCL the corresponding values were 4.8 pmol/L, 5.09 pmol/L, and 3.91 pmol/L, respectively.

When analyzing AMH as a categorical variable according to the respective age group, at 6 months post-treatment, only 27.6% of the HL patients had AMH values below normal for their age, compared to 72.7% of the PMBCL patients (*p* = 0.009). In the ≤30-year age group, only 18.8% of HL patients exhibited impaired ovarian reserve versus 70% of PMBCL patients (*p* = 0.009). Considering the latest time point with available measurements (18 months post-treatment), gonadal dysfunction as indicated by low AMH levels according to age, persisted in 16.7% of HL patients compared to 75% of PMBCL patients (*p* = 0.009). This finding was even more striking for patients younger than 30 years, with 8.3% of HL patients having suppressed AMH values compared to 75% of PMBCL patients (*p* = 0.008). The percentages of HL and PMBCL patients with impaired gonadal function according to the respective age-specific AMH levels over time are shown in Figure 1.

Notably, we observed that from 6 months post-treatment onwards, in HL patients, gonadal function not only did not decline compared to baseline, but AMH actually increased, though not at a statistically significant level: AMH values increased in 76.9% (12 months) and in 83.3% (18 months), compared to pretreatment values in HL patients ≤ 30 years (median AMH pretreatment: 15.87 pmol/L vs. 20.41 pmol/L at 18 months post-treatment; *p* = 0.07).

### 3.5. Secondary Hormonal Markers (FSH, LH, Estradiol, Progesterone)

Serum FSH levels increased significantly during chemotherapy in both HL and NHL cohorts, with a greater magnitude and longer persistence in NHL patients. In HL, median FSH values rose from baseline (FSH0: 4.57 mIU/mL) to mid-treatment (FSH1: 7.2 mIU/mL; *p* < 0.001 vs. baseline) and to peak levels at the end of treatment (FSH2: 7.59 mIU/mL; *p* < 0.001 vs. baseline), with normalization occurring by 12–18 months (FSH12: 6.0 mIU/mL; *p* = 0.057 vs. baseline; FSH18: 6.0 mIU/mL; *p* = 0.079 vs. baseline).

In NHL, FSH increased more sharply (FSH0: 4.1 mIU/mL, FSH1: 6.2 mIU/mL; *p* = 0.09 vs. baseline, FSH2: 6.6 mIU/mL; *p* = 0.03 vs. baseline), remained elevated at 6 months (FSH6: 6.88 mIU/mL; *p* = 0.01 vs. baseline), and normalized by 12 months and 18 months (FSH12: 6.2 mIU/mL; *p* = 0.15 vs. baseline, FSH18: 5.7 mIU/mL; *p* = 0.57 vs. baseline). Importantly, 13% of HL and 40% of NHL patients did not exhibit FSH elevation during treatment. Their FSH levels remained stable and within the normal range throughout treatment and follow-up, but still showed a decline in AMH, indicating the greater sensitivity of AMH in detecting early ovarian injury.

LH levels showed a transient increase during treatment in HL patients (LH0: 6 IU/L to LH1: 9.36 IU/L; *p* = 0.003) but remained stable in NHL patients.

Estradiol and progesterone levels demonstrated minimal fluctuations and remained within normal premenopausal ranges across the study period in both groups. The observed variations were not statistically significant and appeared to reflect physiological hormonal changes associated with the menstrual cycle.

Hormonal measurements for AMH, FSH, LH, estradiol, and progesterone are detailed in Table A1.

### 3.6. Pretreatment AMH Values as a Predictor of Post-Treatment Ovarian Reserve

The pretreatment AMH cut-off of 7 pmol/L was derived from an exploratory post hoc analysis. We identified a pretreatment AMH value of ≥7 pmol/L as an exploratory predictor of preserved post-treatment ovarian reserve following chemotherapy for HL patients. Approximately one-third (29%) of all HL patients and 15% of those younger than 30 years had baseline AMH values of <7 pmol/L. Only 6% of patients with baseline AMH ≥ 7 pmol/L had impaired ovarian reserve at 12 months post-treatment, i.e., lower AMH levels than those appropriate for age, in contrast to 57% among those with AMH < 7 pmol/L; *p* < 0.005. This finding is even more pronounced for female HL patients ≤ 30 years: only 9% of those with baseline AMH ≥ 7 pmol/L showed low 12 months post-treatment AMH levels that were low for their age vs. 100% among those with baseline AMH < 7 pmol/L. For identifying low age-adjusted AMH at 12 months, the <7 pmol/L threshold had a sensitivity of 80.0% (4/5; 95% CI, 28.4–99.5%) and specificity of 84.2% (16/19; 95% CI, 60.4–96.6%). ROC analysis of continuous pretreatment AMH yielded an AUC of 0.853 (95% CI, 0.644–1.000). Because it was identified using the present dataset, it should be regarded as hypothesis-generating rather than as a validated clinical cut-off.

In contrast, regardless of age, pretreatment AMH values in PMBCL patients did not correlate with post-treatment gonadal outcomes and were not predictive of post-treatment ovarian reserve. The majority of patients (75%), regardless of their initial ovarian reserve, had persistently low AMH levels at all time points up to 18 months during the follow-up period, indicating that the chemotherapy regimen was the main determinant of ovarian damage.

Considering the fact that, for a female, having AMH values within the normal range for her age does not necessarily mean that she is fertile, we analyzed our data setting a post-treatment AMH value of 7 pmol/L as an endpoint, designating this value as an exploratory marker of ovarian reserve recovery. Thus, 71% of all HL patients and 92% of those aged ≤ 30 years had AMH values ≥ 7 pmol/L at 18 months after the end of treatment. The corresponding value for PMBCL was 25% both overall and among those aged ≤30 years.

In HL, there was a strong association between the pre- and post-treatment AMH values at the threshold of 7 pmol/L: in patients ≤ 30 years, all patients with pretreatment AMH ≥ 7 pmol/L maintained the endpoint of 12 months post-treatment AMH ≥ 7 pml/L, vs. 50% of those with baseline AMH < 7 pmol/L.

### 3.7. Menstrual Outcomes After Chemotherapy

In HL, 85% of patients reported regular and 15% irregular menstrual cycles at baseline. Amenorrhea occurred in 72% during treatment, while 28% maintained menstruation throughout. Specifically, amenorrhea developed in 11% of patients within the first month, 28% within the second, 17% within the third, and 15% within the first six months of therapy. At one year post-chemotherapy, 66% had resumed normal cycles, and in 34% remained irregular; among those who developed amenorrhea, menstruation returned in 52% within three months, 8% within six months, and 6% after one year, whereas 6% remained amenorrheic.

In NHL, 90% of patients reported normal and 10% reported irregular cycles at baseline. After therapy, 65% maintained normal cycles, while 35% reported amenorrhea or menstrual irregularities. No changes were observed in FL patients treated with bendamustine. Among those receiving R-DA-EPOCH, all developed menstrual irregularities: 42% resumed menses within three months, 25% at six months, 16% at one year, and 17% remained amenorrheic. In patients treated with R-CHOP, all experienced a transient reduction in menstrual frequency lasting two months, followed by resumption of normal cycles in 100% within six months. Menstrual outcomes are detailed in Table 3.

## 4. Discussion

This single-institution prospectively designed study aimed to investigate the impact of chemotherapy on young female patients with lymphoma. Its novelty relies on consecutive measurements at preset time points, allowing the assessment of gonadal function kinetics, not only in the long term but also during chemotherapy and shortly thereafter. Previous research, particularly from the GHSG, has primarily focused on long term outcomes such as birth rates or hormonal status assessed at least a year after treatment [12,13]. Our approach provides a detailed understanding of how disease and/or chemotherapy affects ovarian function over time, and our findings may have significant implications for future fertility and contraceptive planning, since there are no available data concerning contraception during chemotherapy.

The results of the present study first demonstrate that AMH represents the most sensitive marker of ovarian damage in chemotherapy-treated premenopausal women. FSH has traditionally been used to define ovarian insufficiency at different cut-offs ranging between 25 IU/L and 40 IU/L [16]. Compared to FSH, AMH levels decreased earlier after the initiation of chemotherapy and rebounded earlier after the end of treatment, predicting ovarian recovery in HL patients. AMH followed a more consistent pattern of kinetics, as all patients follow the same gradual decrease in AMH values during treatment and a gradual recovery afterwards. Conversely, the same is not evident for serum FSH values, which tend to fluctuate in an unpredictable manner. The reason for this difference in kinetics between AMH and FSH could be that FSH is affected by the menstrual cycle, the administration of GnRH analogues or the use of oral contraceptives, whereas AMH is a hormone that exhibits relative stability and remains unaffected even when prophylactic gonadal treatment is administered [17]. This suggests that AMH may serve as a more sensitive biomarker compared to FSH for tracking changes in ovarian function during and after chemotherapy.

AMH is expressed after birth in ovarian granulosa cells of primary and growing follicles and plays a crucial role in regulating the growth of ovarian follicles [18]. Serum AMH levels are considered to be a powerful predictive marker of ovarian reserve in healthy women and strongly correlate with the number of growing antral follicles without directly assessing oocyte quality, spontaneous conception, clinical pregnancy, or live birth [18]. Therefore, the present findings should be interpreted as changes in ovarian reserve rather than as direct evidence of preserved or impaired fertility [19]. The main advantage of AMH as a marker is that it is independent of the menstrual cycle and is relatively stable [17].

Second, our findings reveal that gonadal function is significantly affected during chemotherapy, though the effects and recovery trajectories differ significantly between HL and PMBCL.

In HL, our data are in accordance with the literature. In both the HD13 and HD14 trials, the investigators reported normal AMH levels measured ≥1 year after 2–4 cycles of ABVD in women < 30 years, while older survivors displayed suboptimal levels [11,12]. Furthermore, they indicated that treatment with two cycles of BEACOPP-escalated followed by two cycles of ABVD had a more detrimental effect on ovarian reserve, as evidenced by reduced AMH and elevated FSH levels [12]. From our data, it is evident that female patients with HL experience a recovery of their gonadal function starting from six months after 4–6 cycles of ABVD. At one year post-treatment, median AMH and FSH levels have normalized. We additionally analyzed AMH according to normal values for age and found that at 18 months post-treatment, only 16.7% of all HL patients and 8.3% of those ≤30 years had impaired gonadal function as indicated by low AMH levels according to age. As far as the menstrual cycle is concerned, 90% of survivors treated for early-stage HL in the HD13 trial reported resuming regular menstrual cycles after therapy, with most achieving this within one year, whereas in advanced-stage disease in the HD15 trial, time to resumption of menstruation took longer than one year and was closely associated with age, with a cut-off 30 years. In our dataset, the median time for menstrual resumption was three months post-treatment in 80% of the participants. Our findings regarding menstrual recovery are broadly consistent with those of the Italian multicenter Ferty Care study, which reported restoration of regular menstrual cycles in 70% of women following lymphoma treatment [20].

A third and surprising finding from our analysis is the fact that baseline AMH levels differed significantly between HL and NHL patients. Thus, at baseline, before treatment, 21% of HL patients had lower AMH values than expected for their respective age group compared to 4% of patients with all other diagnoses. This difference was even more striking for patients ≤ 30 years of age, where 27% of HL patients had subnormal AMH levels for their age group, versus none of the other patients, indicating pre-existing reduced ovarian reserve in HL. To our knowledge, such an observation has not yet been reported and needs further confirmation. Moreover, we observed that in HL patients, there was a trend toward gonadal function improvement after chemotherapy in the majority of patients with a median AMH of 20.41 pmol/L at 18 months post-treatment vs. 15.87 pmol/L at baseline in patients ≤ 30 years. We do not have a clear explanation for this finding, since age and all other characteristics did not differ significantly between the two groups. However, corroborating our results, McLaughlin et al. reported an increase in the number of ovarian follicles by ultrasound after ABVD chemotherapy [21]. These findings suggest a possible direct impact of the disease itself on ovarian reserve. Whether the unique biology of HL, characterized by inflammation, an immune-cell tumor microenvironment, and an immunosuppressive cytokine milieu, may play a role in the suppression of gonadal function, needs exploration [22]. There are reports indicating that male patients with advanced-stage HL and the presence of B symptoms already have oligospermia at diagnosis, before any treatment initiation [23]. However, it is not known whether this extends to the female gender.

The fourth important finding of this study is the detrimental effect of R-DA-EPOCH on ovarian function. Thus, in contrast to patients with HL, patients with PMBCL started with a normal baseline gonadal function, but they experienced prolonged ovarian insufficiency, extending to at least 18 months after the end of treatment, since 75% of the patients with PMBCL still had suboptimal AMH values according to their age group. Even for the patients ≤ 30 years, the median AMH at 18 months was 3.91 pmol/L for PMBCL, significantly lower than 20.41 pmol/L for the HL patients. It is obvious that R-DA-EPOCH chemotherapy, which was homogeneously used in this patient population, could be a probable causative factor for ovarian insufficiency in PMBCL. Considering that R-CHOP used in DLBCL was not associated with such a detrimental effect on ovarian reserve, the observed reduction in ovarian reserve may reflect the combined effects of etoposide, cyclophosphamide, doxorubicin, treatment intensity, and cumulative drug exposure. The limited number of patients precluded subgroup analysis regarding the effect of cumulative doses of etoposide on gonadal function. Our data suggest a more detrimental effect of this regimen on ovarian function compared with that reported in the literature. Ovarian function appears to be preserved in female PMBL patients receiving DA-EPOCH-R, particularly in those younger than 40 years, with the risk of premature menopause seemingly confined to patients over 40 years of age. However, this study did not assess AMH variability, the most important marker of ovarian reserve [24]. Future analyses including more subjects and longer follow-up are needed. Our findings are of utmost importance, since R-DA-EPOCH is the preferred regimen by many institutions, including ours, based on its superiority over R-CHOP21 and the minimization of the need for mediastinal radiotherapy [25,26]. However, serious sequelae have recently been recognized after R-DA-EPOCH, such as secondary AML specifically in female patients and now subfertility [27]. We do not know whether ovarian function will recover with longer follow-up, but hormonal stimulation and laparoscopy necessary for oocyte/ovarian tissue cryopreservation before chemotherapy are usually not feasible in PMBCL patients, even in the best-equipped health facilities, due to the aggressiveness of the disease, frequently necessitating immediate initiation of immunochemotherapy.

Lastly, an exploratory finding of the present study is the identification of a pretreatment AMH level that can predict ovarian reserve in HL patients, especially those ≤30 years old. The pretreatment cut-off of 7 pmol/L was associated with patients who experienced ovarian insufficiency. The cut-off of 7 pmol/L was selected as it best stratified patients according to ovarian reserve based on statistical analyses. Only 9% of HL patients ≤ 30 years with baseline AMH ≥ 7 pmol/L showed low ovarian reserve for their age at 12 months post-treatment vs. 100% among those with baseline AMH < 7 pmol/L, who, however, represented the minority (15% of the young HL population). This finding is in accordance with a study including 124 women with varying etiologies of secondary oligomenorrhea. The investigators identified an AMH threshold of 8 pmol/L (1.12 ng/mL), similar to ours, which demonstrated a sensitivity of 85% and a specificity of 100% for diagnosing ovarian insufficiency in comparison with 26 control subjects [28]. However, differences in the study population and outcome definition preclude direct comparison, and this finding should not be considered an external validation of the threshold identified in our cohort. This cutt-off could contribute to future risk stratification in young HL patients before treatment. As highlighted in a recent systematic review, pretreatment AMH may play an important role in guiding individualized fertility preservation counselling and decision-making in women with cancer [29]. As already stated, ovarian function recovers in the vast majority of HL patients after ABVD chemotherapy. BEACOPP-escalated pioneered by the GHLSG has yielded excellent results in advanced HL and is used by many treating physicians in Europe. However, it is associated with permanent ovarian insufficiency, especially if given for six cycles, among other long-term sequelae [11]. Nowadays, new combinations, such as brentuximab-vedotin/AVD and more recently nivolumab/AVD represents less toxic and equally effective treatment choices. The BrECADD regimen, introduced to replace the conventional BEACOPP-escalated, shows gonadal function recovery and fertility outcomes comparable to those observed in women treated with ABVD [30]. In light of this evolving change in the treatment of advanced HL, our results indicate that gonadal function is generally preserved with ABVD-like regimens, and there is no need for cumbersome procedures for this vulnerable female patient population. However, for the minority with pretreatment AMH levels < 7 pmol/L, ovarian preservation procedures should be strongly recommended and discussed with the patient. Apparently, such a threshold is not applicable in PMBCL, since the vast majority of patients experience severe gonadal injury due to treatment, irrespective of age and pretreatment gonadal function. External validation in larger independent cohorts is required.

Another underestimated issue is consultation about contraceptive measures for women undergoing chemotherapy for lymphoma. Our data suggest that a functional “menopause” occurs during chemotherapy, as indicated by undetectable AMH, elevated FSH levels, and oligo/amenorrhea, observed in 72% of HL patients and 100% of patients receiving R-DA-EPOCH. This raises a question about the role of contraceptives during this period, which is particularly significant given that oral contraceptives can be an additional risk factor for thrombosis, alongside the lymphoma itself. Given that GnRHa administered for ovarian protection has been proven to be more efficient than oral contraceptives, such an alternative regimen should be considered [31]. However, the role of GnRH analogues remains questionable, as emerging data suggest they do not protect against chemotherapy-induced gonadotoxicity [32]. Comprehensive guidance on contraception remains limited and should be based on individual patient circumstances and treatment regimens.

This study has several limitations. First, it was conducted at a single center and included a modest overall sample size, with particularly small subgroups at later assessments. Furthermore, follow-up was limited to 18 months, particularly restricting conclusions regarding long-term ovarian recovery after R-DA-EPOCH. An important limitation is the absence of multivariable adjustment. Residual confounding by age, disease stage, radiotherapy, GnRH analogue use, and other treatment-related factors cannot be excluded, and the observed associations should not be interpreted as independent or causal. Although age was partly addressed through stratified analyses and age-adjusted AMH reference ranges, these approaches do not replace formal multivariable adjustment. Larger studies with sufficient numbers of outcome events are required to confirm these findings using appropriately adjusted models. Given the small sample size, we opted for a conservative approach and did not fit multivariate models.

The non-random and unequal administration of GnRH analogues represents a potential confounding factor, as these agents were used more frequently in patients with NHL than in those with HL (44% vs. 12%). Because the exposed subgroups were small, the independent effect of GnRH analogue use on ovarian recovery could not be reliably evaluated. Although their greater use in the NHL group is unlikely to explain the poorer AMH recovery observed in this group, a potential effect on hormonal or menstrual outcomes cannot be excluded. Although previous research has combined hormonal measurements with antral follicle count (AFC), logistical difficulties in arranging serial ultrasound examinations in our routine clinical setting prevented systematic longitudinal AFC assessment, representing an additional limitation of the present study [20]. Finally, the pretreatment AMH threshold of 7 pmol/L was identified through a post hoc, data-driven analysis and evaluated in the same small cohort, without internal or external validation. It should therefore be regarded as hypothesis-generating and not as a validated clinical decision threshold.

Young women with HL or PMBCL should receive pretreatment counselling regarding reproductive risks, with timely specialist referral for those interested in or uncertain about fertility preservation. Decisions should incorporate age, baseline ovarian reserve, reproductive goals, the planned chemotherapy regimen and treatment urgency.

In our cohort, AMH recovery in HL, treated predominantly with ABVD, contrasted with more persistent suppression following R-DA-EPOCH. Although limited by small subgroup sizes, these findings suggest that women receiving R-DA-EPOCH may warrant particular attention to fertility preservation options and closer follow-up. Increasing age and reduced baseline ovarian reserve also warrant individualized assessment. Oocyte or embryo cryopreservation should be discussed, with ovarian tissue cryopreservation considered when appropriate and feasible; GnRH analogues should not replace established preservation methods [33]. Follow-up should include menstrual and hormonal assessment, with specialist evaluation of persistent abnormalities [33,34]. Neither AMH recovery nor menstrual resumption establishes preserved fertility, and our exploratory 7 pmol/L threshold should not be used as a clinical decision threshold.

Further research is needed to explore the differential impact of various chemotherapy regimens on ovarian function and to validate these findings in larger cohorts. Longitudinal studies are essential in PMBCL in order to investigate whether and when gonadal function recovers after R-DA-EPOCH chemotherapy. Moreover, the AMH threshold of 7 pmol/L as a predictor of persistent diminished ovarian reserve in a large cohort of female HL patients needs to be validated.

## 5. Conclusions

In conclusion, our study first demonstrates that AΜH is the most sensitive and reliable marker of gonadal function, with an exploratory threshold of 7 pmol/L predicting impaired ovarian reserve in HL patients. Second, HL itself may contribute to gonadal dysfunction, possibly through inflammation, and treatment with ABVD may actually restore ovarian reserve, at least in young patients. Thirdly, R-DA-EPOCH, although associated with excellent disease control, has detrimental effects on fertility, at least up to 18 months post-treatment. Moreover, the findings of this study underscore the necessity for continued research to support evidence-based decisions and interventions. Such efforts are vital to ensure that survivors transition to fulfilling lives with comprehensive care that addresses both their cancer treatment and long-term reproductive health.

## Figures and Tables

**Table 1:** Patient baseline characteristics.

Characteristic	HL n = 53	NHL n = 28
Median age, years (range)	28 (18–40)	29 (17–40)
		PMBCL: 28.5 (17–40)
		DLBCL 35 (19–40)
		FL 31 (25–35)
		BL 19
Histological type, n (%)		
	Nodular sclerosis:41 (80%)	PMBCL: 16 (57%)
	Mixed cellularity: 1 (2%)	DLBCL: 9 (32%)
	Lymphocyte rich: 1 (2%)	FL: 2 (7%)
	Unclassified: 8 (16%)	BL: 1 (4%)
Clinical stage, n (%)		
I	1 (2%)	1 (4%)
II	39 (74%)	15 (54%)
III	4 (8%)	4 (14%)
IV	9 (17%)	8 (29%)
B symptoms, n (%)	17 (32%)	8 (29%)
Treatment cycles, n (%)		
4	2 (4%)	0 (0%)
6	51 (96%)	28(100%)
Treatment type, n (%)	47 (89%) ABVD	16 (57%) RDAEPOCH
	6 (11%) BV-AVD	9 (32%) RCHOP
		2 (7%) R/G BENDA
		1 (4%) BL protocol
Extranodal disease, n (%)	13 (26%)	7 (26%)
Radiation therapy, n (%)	7 (14%)	2 (7%)
Menstrual cycle (before treatment initiation), n (%)		
Normal	45 (84%)	24 (86%)
Abnormal	8 (16%)	4 (14%)
Parenthood before, n (%)	12 (24%)	6 (22%)
Oral contraception, n (%)	2 (4%)	0 (0%)
GnRH analogues, n (%)	6 (12%)	12 (44%)
Cryopreservation, n (%)	15 (30%)	3 (12%)

Note: Data for age are depicted as median (range: min–max). Abbreviations: HL: Hodgkin lymphoma; NHL: non-Hodgkin lymphoma; PMBCL: primary mediastinal B-cell lymphoma; DLBCL: diffuse large B-cell lymphoma; FL: follicular lymphoma; BL: Burkitt lymphoma; ABVD: adriamycin, bleomycin, vinblastine, and dacarbazine; BV-AVD: brentuximab vedotin, adriamycin, vinblastine, and dacarbazine; RDAEPOCH: rituximab, dose-adjusted etoposide, vincristine, adriamycin, and cyclophosphamide; RCHOP: rituximab, cyclophosphamide, doxorubicin, vincristine, and prednisone; R/G Benda: rituximab/obinutuzimab and bendamustine.

**Table 2:** Time of hormonal measurement within the prespecified sampling window.

	T0	T1	T2	T6	T12	T18
Total	n = 81	n = 64	n = 54	n = 40	n = 40	n = 30
HL	n = 53	n = 42	n = 35	n = 22	n = 22	n = 21
PMBCL	n = 16	n = 13	n = 12	n = 12	n = 7	n = 4
Other NHL	n = 12	n = 9	n = 7	n = 6	n = 11	n = 5

Note: T0 = baseline, T1 = mid-treatment, T2 = end of treatment, T6 = 6 months post-treatment, T12 = 12 months post-treatment, T18 = 18 months post-treatment, no participants were lost to follow-up. Abbreviations: HL: Hodgkin lymphoma, NHL: non-Hodgkin lymphoma, PMBCL: primary mediastinal B-cell lymphoma.

**Table 3:** Menstrual outcomes following chemotherapy.

REGIMEN	ABVD-like, n/N(%)	RDAEPOCH, n/N(%)
Menstrual irregularities following chemotherapy	38/53 (72%)	15/15 (100%)
Resumption of menses		
3 months	28/53 (52%)	6/15 (40%)
6 months	4/53 (8%)	4/15 (27%)
1 year	3/53 (6%)	2/15 (13%)
Persistent amenorrhea	3/53 (6%)	3/15 (20%)
Treatment-specific findings		Bendamustine: no menstrual changes (100%); R-CHOP-21: frequency irregularities with 100% recovery ≤6 months

## Data Availability

All data generated or analyzed during this study are included in this published article. The data that support the findings of this study are available from the corresponding author upon reasonable request.

## Abbreviations

The following abbreviations are used in this manuscript:

HL	Hodgkin lymphoma
NHL	non-Hodgkin lymphoma
PMBCL	primary mediastinal B-cell lymphoma
DLBCL	diffuse large B-cell lymphoma
FL	follicular lymphoma
BL	Burkitt lymphoma
FSH	follicle-stimulating hormone
LH	luteinizing hormone
AMH	anti-Müllerian hormone
ABVD	(adriamycin, bleomycin, vinblastine, dacarbazine
BV-AVD	brentuximab vedotin, adriamycin, vinblastine, dacarbazine
Beacopp	bleomycin, etoposide, adriamycin, cyclophosphamide, vincristine, procarbazine, prednisolone
RDAEPOCH	rituximab, dose-adjusted etoposide, vincristine, adriamycin, cyclophosphamide
RCHOP	rituximab, cyclophosphamide, doxorubicin, vincristine, prednisone
R/G Benda	rituximab/obinutuzimab and bendamustine

## Appendix A

This table summarizes median serum levels of reproductive hormones (AMH, FSH, LH, estradiol, and progesterone) at six distinct time points from baseline to 18 months post-treatment in patients with HL and NHL. For each time point, *p*-values indicate statistical significance of changes compared to baseline levels.

**Table A1:** Temporal evolution of reproductive hormones and statistical significance in HL vs. NHL. Data are presented as median (n), where n indicates the number of patients with available hormone measurements at each time point.

Group (No Patients)	T0	T1	T1 (p vs. t0)	T2	T2 (p vs. t0)	T6	T6 (p vs. t0)	T12	T12 (p vs. t0)	T18	T18 (p vs. t0)
AMH-HL	10.6 (53)	1.05 (42)	<0.001	2.82 (35)	<0.001	9.92 (22)	0.10	9.90 (22)	0.84	12.33 (21)	0.25
AMH-NHL	15.7 (28)	0.18 (22)	<0.001	0.22 (19)	0.001	4.51 (18)	0.001	5.09 (18)	0.007	7.73 (9)	0.018
AMH-PMBCL	15.83 (16)	0.16 (13)	0.002	0.22 (12)	0.003	4.51 (12)	0.005	1.32 (7)	0.028	3.91 (4)	0.109
FSH-HL	4.57 (53)	7.2 (42)	<0.001	7.59 (35)	<0.001	6.27 (22)	0.020	6.0 (22)	0.057	6.0 (21)	0.079
FSH-NHL	4.1 (28)	6.2 (22)	0.09	6.6 (19)	0.03	6.88 (18)	0.01	6.2 (18)	0.15	5.7 (9)	0.57
FSH-PMBCL	4.2 (16)	8.2 (13)	0.27	7.5 (12)	0.05	7.97 (12)	0.021	5.75 (7)	0.173	6.1 (4)	0.593
LH-HL	6.0 (53)	9.36 (42)	0.003	7.0 (35)	0.011	7.23 (22)	0.65	6.5 (22)	0.458	7.26 (21)	0.332
LH-NHL	7.0 (28)	4.4 (22)	0.638	2.7 (19)	0.776	6.24 (18)	0.647	6.8 (18)	0.814	5.9 (9)	0.779
LH-PMBCL	6.9 (16)	0.3 (13)	0.638	2.7 (12)	0.790	7 (12)	0.929	6.83 (7)	0.249	6.59 (4)	0.593
Estradiol-HL	69.1 5 (53)	70.23 (42)	0.83	69.63 (35)	0.27	126.0 (22)	0.013	105.5 (22)	0.346	99.98 (21)	0.765
Estradiol-NHL	64.32 (28)	9.35 (22)	0.02	29.24 (19)	0.955	64.27 (18)	0.811	103.4 (18)	0.239	111 (9)	0.123
Estradiol-PMBCL	46.88 (16)	5.21 (13)	0.002	8.75 (12)	0.328	60.25 (12)	0.929	137.9 (7)	0.046	153.7 (4)	0.109
Progesterone-HL	0.28 (53)	0.28 (42)	0.22	0.26 (35)	0.36	0.3 (22)	0.87	0.37 (22)	0.21	0.27 (21)	0.79
Progesterone-NHL	0.22 (28)	0.11 (22)	0.008	0.19 (19)	0.427	0.29 (18)	0.603	0.45 (18)	0.754	1.22 (9)	0.889
Progesterone-PMBCL	0.18 (16)	0.07 (13)	0.016	0.18 (12)	0.790	0.36 (12)	0.333	3.46 (7)	0.116	0.71 (4)	0.1

Abbreviations: HL: Hodgkin lymphoma, NHL: non-Hodgkin lymphoma, PMBCL: primary mediastinal B-cell lymphoma, FSH: follicle-stimulating hormone, LH: luteinizing hormone, AMH: anti-Müllerian hormone.
